# Selective Removal of Hexavalent Chromium by Novel Nitrogen and Sulfur Containing Cellulose Composite: Role of Counter Anions

**DOI:** 10.3390/ma16010184

**Published:** 2022-12-25

**Authors:** Xiong Peng, Shujun Liu, Zhijia Luo, Xiwen Yu, Wanwen Liang

**Affiliations:** 1 School of Chemistry and Chemical Engineering, Guangzhou University, Guangzhou 510006, China; 2 School of Chemistry and Chemical Engineering, South China University of Technology, Guangzhou 510640, China; 3 Institute of Biological and Medical Engineering, Guangdong Academy of Sciences, Guangzhou 510316, China

**Keywords:** cellulose, adsorption, selectivity, hexavalent chromium

## Abstract

Exploiting an adsorbent with superb selectivity is of utmost importance for the remediation of Cr (VI)-laden wastewater. In this work, a novel nitrogen and sulfur functionalized 3D macroporous cellulose material (MPS) was prepared by homogeneous cross-link cellulose and polyvinylimidazole, followed by ion exchange with MoS_4_^2−^. MPS exhibited high removal efficiency at a broad pH range (1.0–8.0) and large adsorption capacity (379.78 mg/g) toward Cr (VI). Particularly, outstanding selectivity with an enormous partition coefficient (1.01 × 10^7^ mL/g) was achieved on MPS. Replacing MoS_4_^2−^ with Cl^−^ and MoO_4_^2−^ led to a sharp decline in adsorption selectivity, demonstrating that MoS_4_^2−^ contributed substantially to the selectivity. Results of FTIR, XPS, and apparent kinetic analysis revealed that Cr (VI) was first pre-enriched on the MPS surface via electrostatic and dispersion forces, and then reacted with MoS_4_^2−^ to generate Cr (III), which deposited on MPS by forming Cr(OH)_3_ and chromium(III) sulfide. This study provides a new idea for designing adsorbents with a superior selectivity for removing Cr (VI) from sewage.

## 1. Introduction

The pervasive application of chromium in the field of industry has resulted in the emission of chromium ions into the hydrosphere [1]. Hexavalent chromium anion (Cr (VI)) is one of the main existing forms in aqueous solution, which possesses high virulence and can create a serious threat to human health through accumulation in the food chain [2]. Cr (VI) can cause nosebleeds, pharyngitis, bronchitis, asthma, allergies, and rashes. Long-term exposure or ingestion of Cr (VI) will lead to changes in genetic material, organ cancer, and even to death [3]. The World Health Organization (WHO) set a criterion that the concentration of chromium ions in potable water should be below 50 ppb [3]. Hence, effective methods must be applied for the elimination of Cr (VI) from wastewater. Membrane separation [4], precipitation [5], ion exchange [6], electrochemical flocculation [7], adsorption [8], and photo-catalysis [9], have been reported to reduce the concentration of chromium ions in sewage to a safe level. Due to the traits of easy operation, high efficiency, and low cost, adsorption is widely used among these methods. The adsorption performance is highly relative to the physicochemical properties of the adsorbent, and selecting suitable adsorptive materials can make the adsorption process superior in separation efficiency. Presently, many adsorptive materials such as carbon material [10], MOF [11], Zero-Valent Iron [12], ore [13], and wasted biomass [14], have been applied to eliminate Cr (VI) from sewage. However, most of the adsorbents showed inferior selectivity, thus resulting in difficult targeted adsorption of Cr (VI) from complex wastewater. Therefore, it is highly warranted to exploit adsorptive materials with superb selectivity for eliminating Cr (VI) ions.

Cellulose is an abundant biopolymer material on earth, and the extraordinary characteristics of being non-toxic, cheap, easily available, renewable, and biodegradable make it a promising adsorbent candidate [15]. Nevertheless, large-scale hydrogen bond networks, high crystalline degree, and a supramolecular structure in cellulose lead to the low adsorption capacity of cellulose and thus limit its application in wastewater treatment. Various auxiliary ingredients were used to configure the surface properties to strengthen the adsorption performance. Electrostatic attraction is crucial for Cr (VI) adsorption, and increasing the positive charge density on the cellulose surface is an effective method to enhance Cr (VI) removal efficiency. At present, a variety of nitrogen-containing groups have been used to modify cellulose to increase its surface positive charge density, such as the amine group [16], quaternary ammonium [17,18], and imidazolium [19]. Unfortunately, the competitive adsorption of co-existing ions interfered with Cr (VI) adsorption on most nitrogen-containing cellulose materials, thus leading to reduced adsorption efficiency and selectivity.

The introduction of other specific functional groups that can preferentially interact with pollutants is an effective strategy to improve the adsorption selectivity. For example, iron oxide-loaded anion exchange resin was capable of removing arsenic selectively [20]. The immobilized amino cation on the ion exchange resin surface exerted the role of pre-enrichment arsenic ion, while the inorganic metal oxide nanoparticles provided a specific adsorption binding site, thus contributing to high adsorption selectivity. Similarly, hydrated zirconia hydroxide and lanthanum hydroxide loaded quaternary ammonium salts functionalized straws exhibited higher adsorption selectivity for phosphate ions than quaternary ammonium salts functionalized straws [21,22]. Hence, implanting the special third component into the nitrogen-containing adsorption materials is conducive to boosting the adsorption selectivity. Our previous work showed that Cr (VI) was adsorbed on polyvinylimidazole-modified cellulose composite through ion exchange and electrostatic interactions, but the uptake capacity was negatively affected by coexisting anions [23]. If the cellulose composite is implanted with other specific functional groups, the interference effect of coexisting ions would be shielded. Sulfur-containing functional groups, such as organic polysulfide compounds [24], mercapto groups [25], FeS [26], and MoS_4_^2−^ [27,28] showed a good affinity for heavy metal ions and had also been applied for Cr (VI) removal. Given the ability of polyvinylimidazole modified cellulose to pre-enrich Cr (VI) anion through electrostatic effect, we hypothesized that the incorporation of MoS_4_^2-^ into the cellulose composites would result in a functional material capable of selective and efficient separation of Cr (VI) from complex solutions.

In this work, a novel nitrogen and sulfur containing groups functionalized cellulose composite (MPS) was prepared by homogeneous cross-linking combined with an ion-exchange method and applied to selectively remove Cr (VI) ions. The objects of this work were: (1) to prepare and characterize MPS; (2) to analyze the adsorption behavior of Cr (VI) on MPS; and (3) to reveal the inherent mechanisms of Cr (VI) removal by MPS through characterization and apparent kinetic analysis.

## 2. Materials and Methods

### 2.1. Materials

Microcrystalline cellulose (MCC, column chromatography) and N-vinylimidazole (VIM, 99%) were from Chinese National Medicines and Shanghai Macklin Biochemical Technology Corporation Ltd., respectively. Other reagents involved in the chemical experiment, such as epichlorohydrin (ECH, 99%), urea (99%), potassium dichromate (analytically pure), sodium molybdate (99%), and sodium hydroxide (97%) were supplied by Guangzhou Jingke Chemical Glass Instrument (Guangzhou, China). All the chemicals were directly used in the experiment without any purification.

### 2.2. Preparation of MPS

MPS was prepared by combining homogeneous cross-link with ion exchange methods, and the synthesis routine is shown in Figure S1. First, polyvinylimidazole-modified cellulose was fabricated based on our previous studies [23,29]. Briefly, polyvinylimidazole and MCC with mass ratio of 4/3 were cross-linked by 2 mL ECH to obtain polyvinylimidazole modified cellulose (MP-Cl). Second, 0.1 g MP-Cl was immersed in 0.5 g/L (NH_4_)_2_MoS_4_ solution and stirred for 24 h. Lastly, MPS was obtained by distilled water rinsing, and freeze-drying the solid sample. Similarly, MP-Mo was prepared by immersing MP-Cl in sodium molybdate solution.

### 2.3. Characterization of Adsorbents

The morphology of MPS was observed by field emission scanning electronic microscopy (FESEM, HITACHI SU8220, Tokyo, Japan). The functional groups on the surface of MPS were characterized by FTIR spectroscopy (FTIR, Bruker VERTEX 33, Karlsruhe, Germany). The bonding states of elements on MPS were analyzed by X-ray photoelectron spectroscopy (XPS, K-Alpha, Thermofisher Scientific Company, Waltham, MA, USA). Element analyzer (Elementar Vario EL III, Germany) was applied to detect the nitrogen and sulfur content of MPS. The thermal stability of MPS was measured by thermal gravimetric analyzer (NETZSCH TG 209F3, Germany) with N_2_ atmosphere. The thermal treatment temperature was from 30 °C to 800 °C, and the heating rate was 10 °C/min. Raman spectra were recorded on LabRAM Aramis Raman spectrometer (H.J.Y company, France) with the excitation wavelength at 532 nm.

### 2.4. Adsorption Experiments

A constant temperature shaker was applied to carry out the adsorption performance experiments, and the temperatures used for adsorption were 30, 40, and 50 °C. The adsorption isotherms were obtained by vibrating 20 mL Cr (VI) solution of pH 3.0 with 0.02 g MPS for 24 h. The initial concentrations of Cr (VI) ranged from 20 mg/L to 500 mg/L. In the adsorption kinetic experiment, 0.02 g MPS was put into Cr (VI) solution of pH 3.0 with the initial concentration of 100 mg/L, 300 mg/L, and 500 mg/L, respectively. After a certain contact time, the solution was taken out to measure the Cr (VI) ion concentration. The influence of pH on the Cr (VI) adsorption was studied by changing the initial pH values of 100 mg/L Cr (VI) in the range of 1.0–8.0. In the competitive experiments, all the concentrations of coexisting ions were 100 mM, and the initial concentration of Cr (VI) was 100 mg/L. The solution pH values were 3.0, and the adsorption was performed at 30 °C for 24 h. UV-vis spectrophotometer (UV-2450, Shimadzu, Japan) and ICP-OES (Optima 8300, PerkinElmer, Waltham, MA, USA) were applied to measure Cr (VI) and total chromium concentrations, respectively. Adsorption capacity is expressed as:

(1) $$Q_{e}=\frac{\left(C_{0}-C_{e}\right)\times V}{m}\;$$

where *Q_e_* (mg/g) is the equilibrium adsorption capacity; *C*_0_ and *C_e_* (mg/L) are the initial and equilibrium concentrations of Cr (VI) at adsorption time of 24 h, respectively; *V* (L) and *m* (g) are the solution volume and the weight of adsorbent used in adsorption experimental, separately.

## 3. Results and Discussion

### 3.1. Characterization of MPS

**Figure 1:**
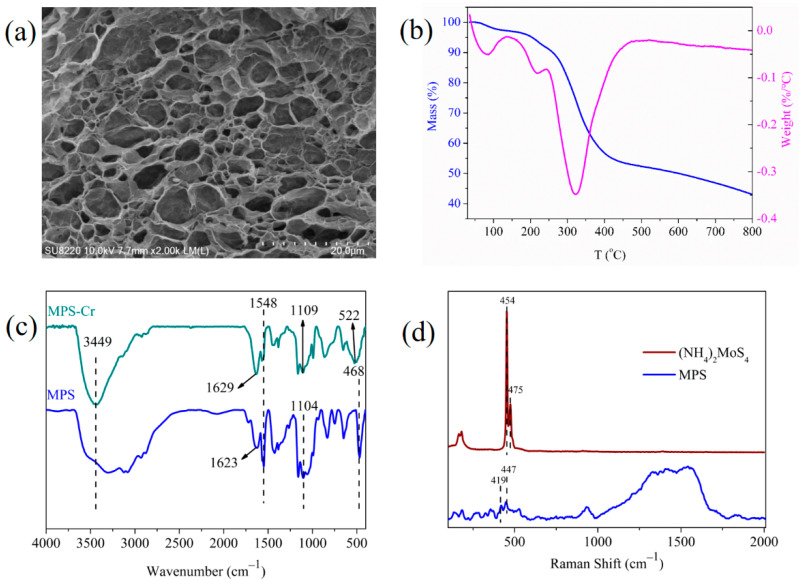
(**a**) SEM image, (**b**) thermogravimetric analysis spectrum of MPS, (**c**) FTIR spectra of MPS before and after adsorption Cr(VI), and (**d**) the Raman spectra of (NH_4_)_2_MoS_4_ and MPS.

The microscale morphology and structure of adsorptive materials are the key factors affecting the adsorption performance. As shown in the FESEM image (Figure 1a), MPS revealed well-defined 3D networks, and the pore size distribution ranged from 2 to 10 μm, indicating MPS possessed a macroporous structure. The stiff cellulose chain and polyvinylimidazole with positive charge served as a strong backbone to support the pore wall and pore expanding reagent, respectively, thus contributing to the formation of a macropore [30]. The macroporous architecture would afford a transport channel and be conducive to the diffusion of Cr (VI) ions.

The thermal analysis spectrum of MPS showed that the quality loss of MPS can be cut into three sections (Figure 1b). The first mass loss stage was from 30 °C to 150 °C, and the mass loss was about 4%, which was ascribed to the removed bound water on the MPS surface. The second stage was 280–400 °C, and the mass loss in this stage was about 40%. The maximum decomposition rate appeared at about 320 °C, and cellulose started to decompose in this stage [31]. At the third stage (400–800 °C), the quality loss of MPS declined slowly, which was due to the decomposition of MoS_4_^2−^ [32].

As shown in Figure 1c, the peak at 1104 cm^−1^ in the FTIR spectrum was the S-S stretching vibration absorption peak, and the broad absorption peak around 468 cm^−1^ was attributed to the Mo-S stretching vibration [33], which illustrated that MoS_4_^2−^ was bonded on the cellulose surface. The characteristic peak of the imidazole cation appeared at 1546 cm^−1^, confirming that MoS_4_^2−^ combined with the imidazole cation through electrostatic action [23,34]. In addition, there was no obvious characteristic peak of the hydroxyl group on cellulose around 3449 cm^−1^, manifesting that the hydroxyl group of cellulose may take part in the reaction of the MPS synthesis process. It was reported that MoS_4_^2−^ formed weak hydrogen bonds with amine groups of antibiotics (Mo-S^…^NH_2_^+^) [35], and hydroxide ions of hydrotalcite (Mo-S^…^HO) [36]. Therefore, it made sense that a new hydrogen bond was formed between MoS_4_^2−^ and hydroxyl groups of cellulose and brought in the change of thehydroxyl characteristic peak at 3449 cm^−1^.

In order to further prove the hydrogen bonding interaction between hydroxyl groups of cellulose and MoS_4_^2−^, Raman spectroscopy analysis was performed on (NH_4_)_2_MoS_4_ and MPS (Figure 1d). The Mo-S tensile vibration absorption peaks of (NH_4_)_2_MoS_4_ appeared at 454 cm^−1^ and 474 cm^−1^ [37], while the peaks on MPS were at 419 cm^−1^ and 447 cm^−1^. The red shift of the Mo-S tensile bond confirmed that cellulose combined with MoS_4_^2−^ in the form of a weak hydrogen bond (Mo-S^…^HO).

**Figure 2:**
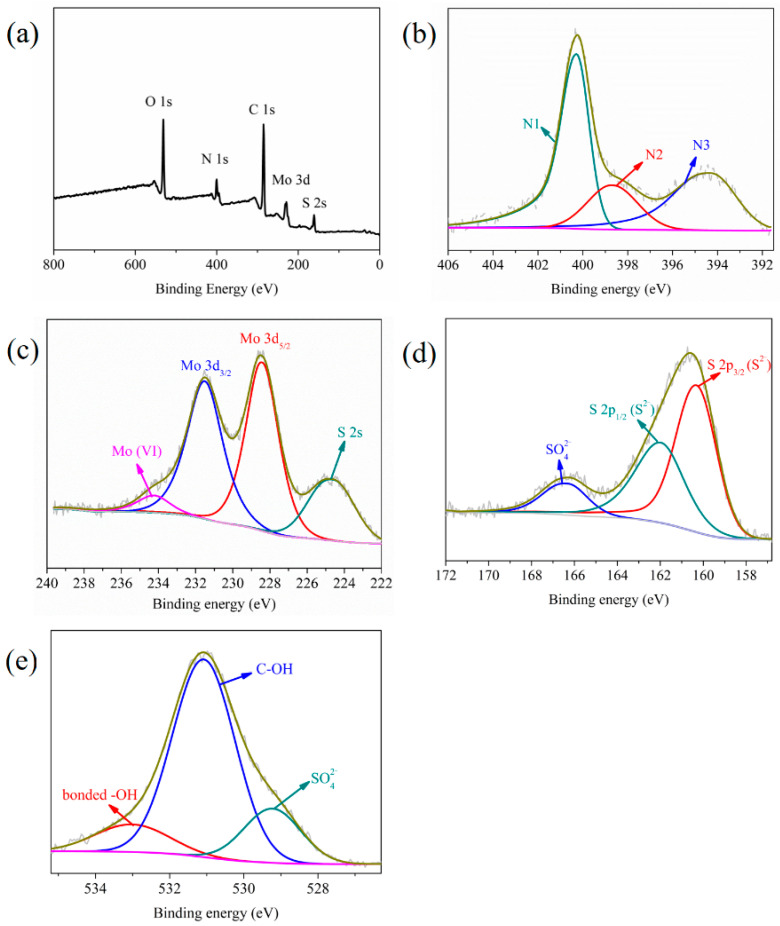
(**a**) XPS spectrum, and high-resolution spectra of MPS: (**b**) N 1s, (**c**) Mo 3d, (**d**) S 2p, and (**e**) O 1s.

The element composition of MPS detected by XPS and CHNS elemental analysis is shown in Table 1 and results reflected that nitrogen and sulfur functional groups were grafted on MPS. The conspicuous characteristic peaks of C, O, N, Mo, and S elements appeared in the XPS spectrum of MPS (Figure 2), while the characteristic peak of the Cl element was not obvious and its content was only 0.55 at %. This phenomenon suggested Cl^−^ underwent ion exchange with MoS_4_^2−^. The high-resolution N 1s spectrum showed that the characteristic peaks of –N^+^=, –N=, and –N< (marked as N1, N2, and N3) appeared at 400.2 eV, 398.7 eV, and 394.3 eV, respectively, indicating that a stable ionic bond was formed between imidazole functional groups and MoS_4_^2-^. In the high-resolution Mo 3d spectrum, the peaks at 228.4 eV and 231.5 eV originated from the spin coupling of Mo 3d_5/2_ and Mo 3d_3/2_ orbitals, respectively. The peaks at 234.2 eV and 224.6 eV were the characteristic peaks of Mo (VI) and S 2s energy orbit, respectively [38]. The peaks with binding energies of 160.0 eV and 161.4 eV deconvoluted from the S 2p spectrum were attributed to the peaks of S 2p_3/2_ and S 2p_1/2_ spin orbits, respectively, revealing that the S element existed in the form of S^2−^. The peak at 166.3 eV was assigned to the characteristic peak of SO_4_^2−^ [39], which accounted for about 11.27% of the total S content, reflecting that part of S^2−^ was oxidized to SO_4_^2−^. The peaks at binding energies of 529.2 eV and 531.4 eV deconvoluted from the O 1s peak were the characteristic peaks of SO_4_^2−^ and C-OH, respectively. The peak at 532.8 eV was the characteristic peak of the hydrogen bond, which also verified that MoS_4_^2−^ interacted with the hydroxyl groups of cellulose through the hydrogen bond. The above analysis showed that MoS_4_^2−^ was anchored on cellulose through ion exchange, electrostatic, and hydrogen bonding interactions.

### 3.2. Adsorption Kinetics

**Figure 3:**
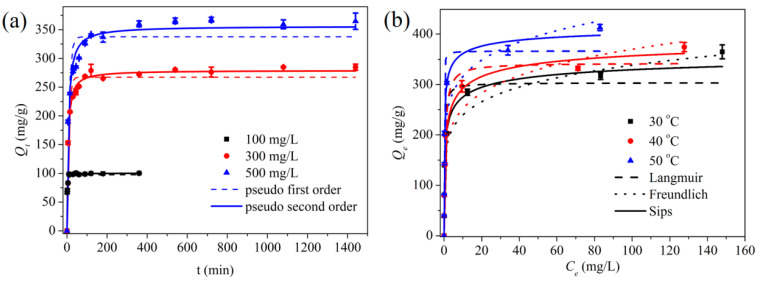
(**a**) Effect of adsorption time on the removal capacity, and (**b**) adsorption isotherms of Cr (VI) on MPS.

The Cr (VI) adsorption amount of MPS affected by adsorption time is illustrated in Figure 3a. All the adsorption curves demonstrated similar tendencies, and the process can be illustrated by two stages: the fast and main removal stage, and the deceleration adsorption stage. The variation of adsorption amount was highly correlated with the pore structure, the number of available adsorption sites, and the driving force of mass transfer. In the initial stage, a large number of effective active sites were conducive to adsorbing Cr (VI). The macroporous structure of MPS and the high Cr (VI) concentration gradient between the bulk solution and MPS surface were favorable to the diffusion of Cr (VI). As the adsorption time prolonged, more and more adsorption sites were occupied, and the steric hindrance and electrostatic repulsion effects made it difficult for the Cr (VI) ions in the solution to contact the remaining active sites of MPS. In addition, the weakening of the driving force for external diffusion also slowed down the adsorption rate. Moreover, the initial concentration of Cr (VI) also displayed an impact on the adsorption rates. When the initial concentration was 100 mg/L, the adsorption rate was fast and the adsorption equilibrium time was about 60 min. As the initial concentration of Cr (VI) increased, the adsorption rate declined and it took about 480 min to reach adsorption equilibrium.

The adsorption data were explained by pseudo first-order and pseudo second-order kinetic models [18,40], and the corresponding kinetics parameters are listed in Table 2. The pseudo second-order kinetic model matched the experimental data better (R^2^ > 0.99), inferring that the adsorption process of Cr (VI) on MPS obeyed the pseudo second-order kinetic model and the entire removal process was controlled by the steps of surface adsorption. The adsorption rate constant (*k*_2_) declined with the rise of initial Cr (VI) concentration, which might be owing to the competitive adsorption between high-concentration Cr (VI) ions for active sites.

### 3.3. Adsorption Isotherms and Thermal Dynamics Analysis

Figure 3b illustrates the influence of adsorption temperature on the removal capacity of MPS. Higher adsorption temperature led to the rise of adsorption capacity, implying that the removal process of Cr (VI) by MPS was endothermic. It is fact that enhanced average kinetic energy of Cr (VI) and redox reactions contributed to the improved adsorption capacity at high temperatures [41]. Langmuir, Freundlich, and Sips’ adsorption isotherm models were used to describe the adsorption behavior [42], and the corresponding calculation parameters are shown in Table 3. The fitting results showed that the Sips isotherm model was more suitable to explain the experimental data, and the elimination of Cr (VI) by MPS was a multi-layer, non-uniform adsorption process. The maximum capacities of Cr (VI) calculated by the Sips model at 30 °C, 40 °C, and 50 °C were 379.78 mg/g, 408.91 mg/g, and 426.63 mg/g, respectively.

The maximum Cr (VI) adsorption capacity of MPS was in comparison with other nitrogen-containing and sulfur-containing adsorbents (Table 4). Comparative analysis implied that MPS exhibits higher adsorption capacity than other nitrogen-containing and sulfur-containing adsorbents, which might be ascribed to the fact that MoS_4_^2−^ and imidazole groups synergistically enhanced the adsorption performance of MPS for Cr (VI).

The calculated thermodynamics parameters are revealed in Table 5. Δ*G* < 0 and Δ*H* > 0 meant the adsorption of Cr (VI) was a spontaneous and endothermic process. ΔS > 0 indicated that the degree of freedom of the solid/liquid interface increased during the adsorption process. All three experimental temperatures showed |TΔ*S*| > |Δ*H*|, manifesting the adsorption of Cr (VI) on MPS was mainly managed by the entropy effect.

### 3.4. Effect of Initial Solution pH

**Figure 4:**
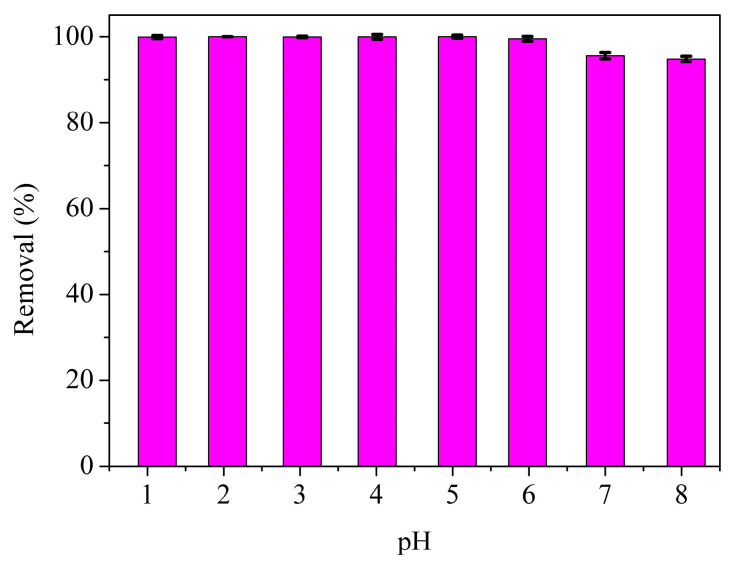
Effect of pH on the Cr (VI) removal efficiency.

The pH value of the solution can simultaneously influence the surface physicochemical properties of the adsorbent and the form of the Cr (VI) ion, thus playing a vital role during the adsorption process. Most adsorptive materials, such as nitrogen-containing adsorbent [48], and MoS_2_ [44], showed the maximum adsorption capacity for Cr (VI) at the pH range from 2.0 to 5.0, due to the strong electrostatic effect between the adsorptive materials and Cr (VI) ion under low pH conditions. When the pH value was below 4, protonated imidazole groups of MPS were in favor of HCrO_4_^−^ adsorption via electrostatic attraction. With the increase of pH, and the decreased positive charge numbers on the surface of MPS, the existence of CrO_4_^2−^ and the interference effect of OH^−^ would bring in the attenuated electrostatic attraction and decline of adsorption efficiency. However, as shown in Figure 4, the removal efficiencies of Cr (VI) on MPS were higher than 95% at the pH range 1.0–8.0, demonstrating MPS had excellent adsorption capacity for Cr (VI) in a wide pH range. These phenomena illustrated that the interaction between MoS_4_^2−^ and Cr (VI) was stronger than the electrostatic attraction. The strong interaction between MoS_4_^2−^ and Cr (VI) would strengthen the adsorption selectivity of Cr (VI).

### 3.5. Adsorption Selectivity

**Figure 5:**
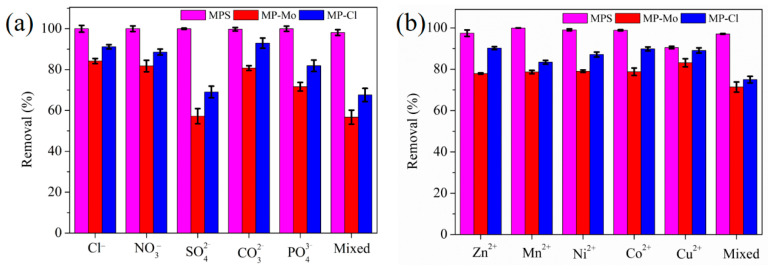
Effect of (**a**) anions, and (**b**) cations on the removal efficiency of Cr (VI).

Figure 5 illustrates the influence of different coexisting ions on the Cr (VI) removal by MPS. The adsorption efficiency was above 92% both in co-existing anions and cations solutions, revealing that MPS exhibited excellent adsorption selectivity for Cr (VI). SO_4_^2−^ has the same tetrahedral structure, charge number, and similar ionic radius as Cr (VI), and it is prone to compete with Cr (VI) for the active sites and display an adverse influence on Cr (VI) adsorption. However, the adsorption efficiency of MPS was almost unaffected by the interference of SO_4_^2−^. This phenomenon was due to the fact that the imidazole cation of MPS promoted the pre-concentration of Cr (VI) through the Donnan membrane effect, and MoS_4_^2−^ underwent strong interaction with Cr (VI), and the two kinds of adsorption forces synergistically enhanced the selective adsorption of Cr (VI). In order to further explore the influence of counter anions of cellulose composites on the adsorption selectivity of Cr (VI), MP-Cl and MP-Mo were applied for selective adsorption of Cr (VI). Results indicated that the adsorption efficiencies of MP-Cl and MP-Mo on Cr (VI) were 67–91% and 56–84%, respectively, and SO_4_^2−^ showed the most adverse impact on Cr (VI) removal.

The distribution coefficient (*K_d_*) was an important index to evaluate the affinity of adsorptive material, and is expressed as:

(2) $$K_{d}=\frac{C_{0}-C_{e}}{C_{e}}\times \frac{V}{m}\;$$

where *C*_0_, *C_e_*, *V*, and *m* are the same with Formula (1). Generally speaking, the material with a *K_d_* value greater than 10^4^ mL/g is an excellent adsorbent [37]. The *K_d_* values of MPS, MP-Cl, and MP-Mo in coexisting ions solutions were 1.26 × 10^4^–1.01 × 10^7^ mL/g, 2.20 × 10^3^–1.29 × 10^4^ mL/g and 1.32 × 10^3^–5.30 × 10^3^ mL/g, respectively, revealing MPS exhibited outstanding adsorption selectivity for Cr (VI). For one thing, the positive charge density on the surface of MP-Cl was higher than that of MP-Mo and MPS, thus MP-Cl could adsorb Cr (VI) more easily through electrostatic interaction. For another, the smaller radius of Cl^-^ made it more prone to undergo ion exchange reaction with Cr (VI) in solution [49]. Hence, MP-Cl should exhibit better selectivity than MP-Mo and MPS. Nevertheless, the case of MPS was opposite to expected, which can be ascribed to the fact that the intensive interaction between MoS_4_^2−^ and Cr (VI) surpassed the electrostatic and ion-exchange interaction, thus endowing MPS superb adsorption selectivity for Cr (VI). Hence, the introduction of a proper counter anion on the cellulose material with the Donnan membrane pre-enrichment effect was one of the effective strategies to achieve high adsorption selectivity of Cr (VI).

### 3.6. Adsorption Mechanisms

#### 3.6.1. Characterization Analysis

**Figure 6:**
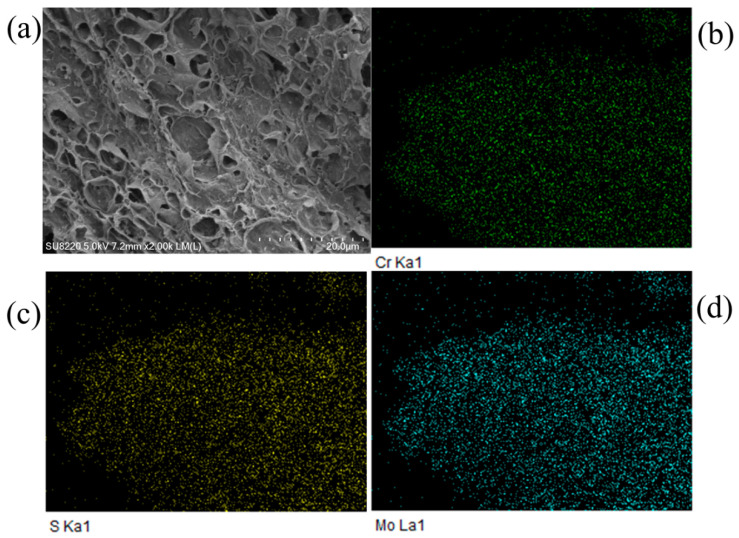
(**a**) SEM image of MPS after adsorption of Cr (VI), and EDS diagram of Cr (VI) loaded MPS: (**b**) Cr, (**c**) S, and (**d**) Mo element distribution.

The FESEM image of Cr (VI) loaded MPS revealed that the apparent morphology of MPS did not change significantly after adsorption (Figure 6). In the EDS spectrum, Cr, Mo, and S elements were uniformly distributed on the surface of MPS with large overlap areas, proving that Cr (VI) underwent a strong chemical interaction with MoS_4_^2^.

As shown in the FTIR spectrum of Cr (VI) loaded MPS (Figure 1c), the vibration peak at 468 cm^−1^ disappeared, suggesting MoS_4_^2−^ was involved in the adsorption process. The S-S stretching vibration bond moved to 1109 cm^−1^, indicating that S^2−^ was oxidized to SO_4_^2−^ during the adsorption process. The absorption peak of hydroxyl stretching vibration at 1623 cm^−1^ shifted to 1629 cm^−1^ and the peak intensity increased, which might be ascribed to the formation of carboxyl groups [23]. At 3449 cm^−1^, the intensity of the hydroxyl absorption peak increased and the peak became wide, this phenomenon can be explained by two aspects. For one thing, the intensive interaction between MoS_4_^2−^ and Cr (VI) destroyed the hydrogen bond between MoS_4_^2−^ and hydroxyl groups, and part of the hydroxyl group was exposed. For another, amorphous Cr (OH)_3_ was formed and deposited on the surface of the adsorbent.

**Figure 7:**
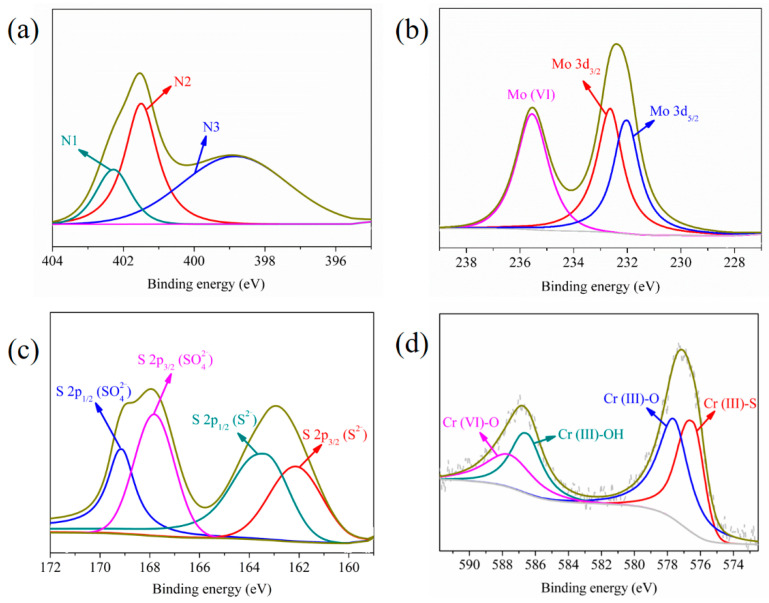
High-resolution XPS spectra of Cr (VI) loaded MPS: (**a**) N 1s spectrum, (**b**) Mo 3d spectrum, (**c**) S 2p spectrum, and (**d**) Cr 2p spectrum.

High-resolution XPS spectra of Cr (VI) loaded MPS are exhibited in Figure 7. N 1s spectrum manifested that the binding energies of N1, N2, and N3 migrated to 402.3 eV, 401.6 eV, and 398.9 eV, respectively, verifying that imidazole groups adsorbed Cr (VI) through electrostatic interaction and dispersion force [50]. In the Mo 3d spectrum, the characteristic peaks of Mo 3d_5/2_ and Mo 3d_3/2_ spin orbits shifted, and the relative content of Mo (VI) increased, while the characteristic peak of S 2s orbital at 224.6 eV disappeared, illustrating the structure of MoS_4_^2−^ had changed. The binding energies of S 2p_3/2_ and S 2p_1/2_ orbits of SO_4_^2−^ appeared at 167.8 eV and 169.1 eV, respectively, and the proportion of SO_4_^2−^ increased to 35.5%, suggesting that S^2−^ was oxidized to SO_4_^2−^ during the adsorption process. Moreover, both Cr (VI) and Cr (III) existed in the Cr 2p spectrum, and the proportion of Cr (VI) was 15.64%, reflecting that most of the adsorbed Cr (VI) was reduced to Cr (III). The peaks at binding energies of 586.5 eV, 577.6 eV, and 576.5 eV were the characteristic peaks of Cr (III)-OH, Cr (III)-O, and Cr (III)-S, respectively, revealing that Cr (III) precipitated on the surface of MPS in the form of Cr(OH)_3_ and chromium(III) sulfide.

#### 3.6.2. Apparent Kinetic Analysis

**Figure 8:**
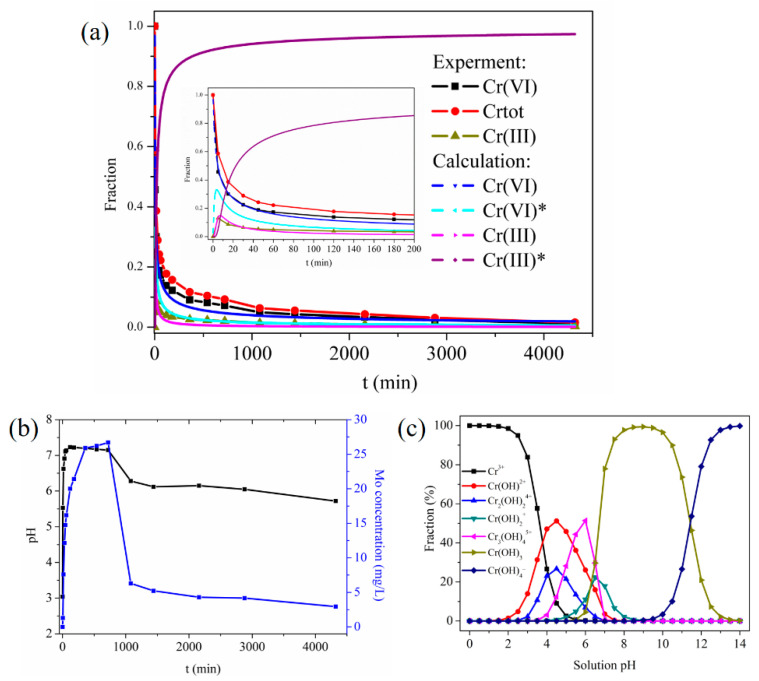
The variation of (**a**) chromium content, (**b**) solution pH value and Mo concentration with time, and (**c**) the form distribution of Cr (III) varied with solution pH value (operation conditions: 200 mg/L Cr (VI) with pH 3.0 at 30 °C).

The apparent kinetic analysis was used to investigate the adsorption process. The relative content of each component in the adsorption process varied with adsorption time as shown in Figure 8a. The content of Cr (VI) and total chromium in the solution showed a downward trend with the proceeding of the adsorption process. The concentration of Cr (III) in the solution rose rapidly within a short period of time (15 min), and then decreased slowly, which indicated that Cr (VI) was rapidly reduced to Cr (III). Figure 8b displays that the pH value of the solution increased sharply to reach a plateau and then gradually decreased. The curve of Mo concentration change in the solution exhibited a similar trend to that of pH value. The oxidation–reduction reaction between Cr (VI) and MoS_4_^2−^ or hydroxyl groups of MPS consumed a large amount of H^+^ in the solution, thus causing the pH of the solution to rise sharply. The species distribution curve of Cr (III) manifested that Cr (III) began to precipitate when the solution pH was above 6.0 (Figure 8c). Therefore, Cr (III) in the solution started to react with OH^–^ and the concentration of Cr (III) in the solution decreased after 15 min. The ion exchange and redox reaction between MoS_4_^2−^ and Cr (VI) led to the release of Mo into the solution in the form of MoS_4_^2−^ and MoO_4_^2−^, and caused the content of Mo in the solution to increase rapidly. As the adsorption-reduction process continued, MoS_4_^2−^ formed a stable complex with Cr (III) in the solution, and resulted in the Mo content in the solution dropping sharply. In addition, MPS also adsorbed MoO_4_^2−^ through electrostatic interaction and contributed to the slow decrease of Mo content.

The above analysis suggested that the removal of Cr (VI) involved three steps: surface adsorption, reduction, and Cr (III) coordination or precipitation. The mechanisms of the adsorption process were described by Formula (3). In the first step, Cr (VI) was adsorbed on the surface of MPS with the rate constant *k*_1_ to form Cr (VI)*. In the second step, part of Cr (VI)* was reduced to Cr (III) at a rate constant of *k*_2_, and part of Cr (III) was released into the solution. In the third step, Cr (III) in the solution was anchored on the surface of the MPS at a rate constant of *k*_3_ to form Cr (III)*.

(3) $$\mathrm{Cr}\;(\mathrm{VI})\to \mathrm{Cr}\;{\left(\mathrm{VI}\right)}^{*}\to \mathrm{Cr}\;(\mathrm{III})\to {\mathrm{Cr}\;(\mathrm{III})}^{*}$$

Assuming that the adsorption reaction orders of these three steps were *n*_1_, *n*_2_, *n*_3_, the apparent kinetics of Cr (VI) adsorption can be expressed as the following four ordinary differential equations:

(4) $$\frac{d_{\mathrm{Cr}\;(\mathrm{VI})}}{d_{t}}{=-k}_{1}{\cdot [\mathrm{Cr}\;(\mathrm{VI})]}^{n_{1}}$$

(5) $$\frac{d_{\mathrm{Cr}\;{\left(\mathrm{VI}\right)}^{*}}}{d_{t}}{=k}_{1}{\cdot [\mathrm{Cr}\;(\mathrm{VI})]}^{n_{1}}\;{-\;k}_{2}\cdot [\mathrm{Cr}\;{\left(\mathrm{VI}\right)}^{*}{]}^{n_{2}}$$

(6) $$\frac{d_{\mathrm{Cr}\;\left(\mathrm{III}\right)}}{d_{t}}{=k}_{2}\cdot [\mathrm{Cr}\;{\left(\mathrm{VI}\right)}^{*}{]}^{n_{2}}{\;-\;k}_{3}\cdot [\mathrm{Cr}\;{\left(\mathrm{III}\right)}^{*}{]}^{n_{3}}$$

(7) $$\frac{d_{\mathrm{Cr}\;{\left(\mathrm{III}\right)}^{*}}}{d_{t}}{=k}_{3}\cdot [\mathrm{Cr}\;\left(\mathrm{III}\right){]}^{n_{3}}$$

In order to facilitate the experiment calculation, all the concentrations were normalized, namely:

(8) $${[\mathrm{Cr}}_{\mathrm{tot}}{]=C}_{t,\mathrm{tot}}{/C}_{0}$$

(9) $${[\mathrm{Cr}}_{\mathrm{tot}}]=\left[\mathrm{Cr}\;\left(\mathrm{VI}\right)\right]+[\mathrm{Cr}\;(\mathrm{III})]$$

Among them, C_t,tot_ was the total chromium concentration in the solution at time *t*, and *C*_0_ was the initial concentration of Cr (VI). When t = 0:

(10) $$[{\mathrm{Cr}}_{\mathrm{tot}}]=\left[\mathrm{Cr}\;\left(\mathrm{VI}\right)\right]=1$$

(11) $${\left[\mathrm{Cr}\;(\mathrm{VI}\right)}^{*}]=\left[\mathrm{Cr}\;\left(\mathrm{III}\right)\right]=[\mathrm{Cr}\;{\left(\mathrm{III}\right)}^{*}]=0\;$$

The fourth-order Runge-Kutta method of the genetic algorithm toolbox in the Matlab software was used to optimize the minimum value of the Formula (12) [51]. In the formula, Cr(VI, ek), and Cr(VI, ck) were the experimental and calculated values of the k-th point of Cr (VI), and *n* was the number of experimental points.

(12) $$\mathrm{OF}=\frac{\sum_{k=1}^{n}((\mathrm{Cr}\left(\mathrm{VI},\mathrm{ek}\right)\;-\;(\mathrm{Cr}(\mathrm{VI},\mathrm{ck})){)}^{2}+\sum_{k=1}^{n}((\mathrm{Cr}\left(\mathrm{III},\mathrm{ek}\right)\;-\;(\mathrm{Cr}\left(\mathrm{III},\mathrm{ck}\right)){)}^{2}}{n}$$

The fitting curves suggested that the simulated values of Cr (VI) and Cr (III) were close to the experimental values, and the correlation coefficients were 0.998 and 0.973, respectively, which further confirmed that the process involved adsorption, reduction, and coordination. The calculated reaction constants *n*_1_, *n*_2_, and *n*_3_ were 2.79, 2.43, and 1.93, respectively, and the rate constants *k*_1_, *k*_2_, and *k*_3_ were 0.32 min^−1^, 0.78 min^−1^, 1.56 min^−1^, respectively. These phenomena indicated that the anchoring of Cr (III) on the surface of MPS was the fastest step, while the adsorption of Cr (VI) was the rate-determining step of the whole process. The implanting of MoS_4_^2−^ reduced the surface positive charge density of the cellulose composite, and weakened the electrostatic attraction, thus leading to a slow adsorption rate of Cr (VI) on MPS.

**Figure 9:**
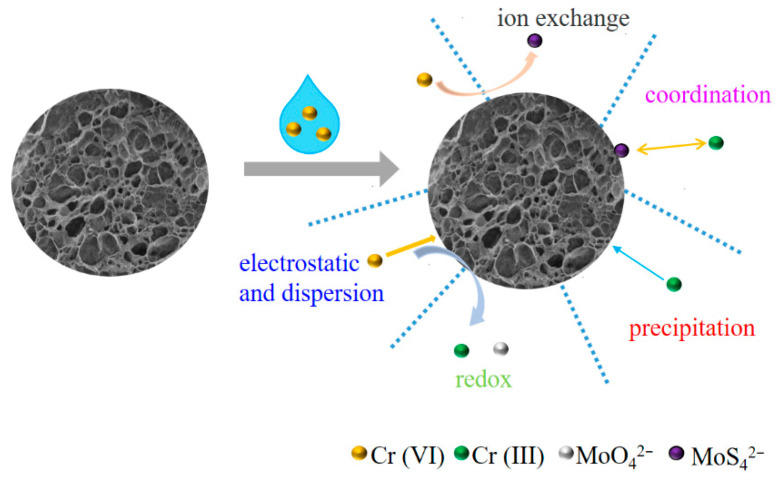
Proposed adsorption mechanisms of Cr (VI) on MPS.

Therefore, the adsorption mechanisms of Cr (VI) on MPS were proposed (Figure 9). MPS first attracted Cr (VI) through electrostatic and weak dispersion interactions, and the adsorbed Cr (VI) underwent ion exchange and redox reaction with MoS_4_^2−^. The Cr (III) ions derived from Cr (VI) reduction were deposited on the surface of MPS through precipitation and coordination. The synergistic effect of imidazole groups and MoS_4_^2−^ endowed MPS with excellent adsorption selectivity for Cr (VI).

## 4. Conclusions

MPS with nitrogen and sulfur groups showed a high adsorption capacity (379.78 mg/g) and selectivity (*K_d_* = 1.01 × 10^7^ mg/L) toward Cr (VI). The adsorption process was a spontaneous and endothermic process, and adsorption isotherm and kinetic models showed that the adsorption process conformed to Sips and pseudo-second kinetics models. The counter anion MoS_4_^2−^ contributed to the superb adsorption performance. The adsorption process involved multiple forces: electrostatic attraction and dispersion force promoted the pre-enrichment of Cr (VI) on MPS, and then adsorbed Cr (VI) underwent reaction with MoS_4_^2−^ and hydroxyl to generate Cr (III), which was deposited on MPS with the species of Cr (OH)_3_ and chromium(III) sulfide. These findings would provide an effective strategy to design an advanced adsorbent with high selectivity for removing Cr (VI) from wastewater.

## Figures and Tables

**Table 1:** Elemental composition of MPS.

Element	XPS (at %)	CHNS (wt %)
C	62.28	32.45
H	-	5.72
N	7.02	7.66
S	6.10	11.73
Mo	1.44	-
Cl	0.55	-
O	22.61	-

**Table 2:** The adsorption kinetics parameters of Cr (VI) on MPS.

Concentration (mg/L)	Pseudo First-Order Model		Pseudo Second-Order Model	
	*k*_1_ (min^−1^)	R^2^	*k*_2_ (g/mg∙min)	R^2^
100	8.72 × 10^−1^	0.95	1.59 × 10^−2^	0.99
300	1.29 × 10^−1^	0.95	7.46 × 10^−4^	0.99
500	1.01 × 10^−1^	0.89	4.31 × 10^−4^	0.97

**Table 3:** The adsorption isotherm models parameters of Cr (VI) on MPS.

Isotherm Models		T (°C)		
		30	40	50
Langmuir	*Q_max,cal_* (mg/g)	303.49	342.83	366.41
	*K_L_* (L/mg)	4.30	1.72	2.46
	R^2^	0.91	0.94	0.87
Freundlich	*K_f_* (mg/g·(L/mg)^1/n^)	169.44	171.94	228.18
	*n*	6.66	6.01	7.80
	R^2^	0.96	0.95	0.86
Sips	*Q_max,cal_* (mg/g)	379.78	408.91	426.63
	*K_s_* (L/mg)	1.15	0.81	5.45
	*n*	2.53	2.28	2.33
	R^2^	0.99	0.98	0.96

**Table 4:** Comparing the adsorption capacity of Cr (VI) with different adsorbents.

Adsorbent	*Q_max_* (mg/g)	Operating Conditions	Reference
Thiol-modified cellulose nanofibrous	87.50	22 °C, 1 h	[25]
Fe-MoS_4_	69.81	30 °C, 3 h	[32]
LDHs@MoS_2_	76.30	25 °C, 24 h	[43]
PVP/MoS_2_	142.24	25 °C, 24 h	[44]
Fe_3_O_4_/MCC-PEI	198.80	30 °C, 2 h	[45]
Ppy–Fe_3_O_4_/rGO	293.30	30 °C, 12 h	[46]
MoS_2_@PANI/PAN	335.04	25 °C, 24 h	[47]
MPS	379.78	30 °C, 24 h	This work

**Table 5:** Thermodynamic parameters of Cr (VI) adsorption on MPS.

Δ*H* (kJ/mol)	Δ*S* (kJ/(mol·K))	T (K)	Δ*G* (kJ/mol)
5.19	0.09	303.15	−22.06
		313.15	−22.96
		323.15	−23.86

## Data Availability

Data sharing is not applicable to this article.

## Supplementary Materials

The following supporting information can be downloaded at: https://www.mdpi.com/article/10.3390/ma16010184/s1, Figure S1: The synthesis routine of MPS composite.

## References

[1] Blanes P.S., Bordoni M.E., González J.C., García S.I., Atria A.M., Sala L.F., Bellú S.E. (2016). Application of soy hull biomass in removal of Cr(VI) from contaminated waters. Kinetic, thermodynamic and continuous sorption studies. J. Environ. Chem. Eng..

[2] Wan J., Liu F., Wang G., Liang W., Peng C., Zhang W., Lin K., Yang J. (2021). Exploring different mechanisms of biochars in removing hexavalent chromium: Sorption, reduction and electron shuttle. Bioresour. Technol..

[3] Rakhunde R., Deshpande L., Juneja H.D. (2012). Chemical Speciation of Chromium in Water: A Review. Crit. Rev. Environ. Sci. Technol..

[4] Gebru K.A., Das C. (2018). Removal of chromium (VI) ions from aqueous solutions using amine-impregnated TiO_2_ nanoparticles modified cellulose acetate membranes. Chemosphere.

[5] Dhal B., Thatoi H.N., Das N.N., Pandey B.D. (2013). Chemical and microbial remediation of hexavalent chromium from contaminated soil and mining/metallurgical solid waste: A review. J. Hazard. Mater..

[6] Pakade V.E., Tavengwa N.T., Madikizela L.M. (2019). Recent advances in hexavalent chromium removal from aqueous solutions by adsorptive methods. RSC Adv..

[7] Barrera-Díaz C.E., Lugo-Lugo V., Bilyeu B. (2012). A review of chemical, electrochemical and biological methods for aqueous Cr(VI) reduction. J. Hazard. Mater..

[8] Mortazavian S., Murph S.E.H., Moon J. (2022). Biochar Nanocomposite as an Inexpensive and Highly Efficient Carbonaceous Adsorbent for Hexavalent Chromium Removal. Materials.

[9] Baaloudj O., Nasrallah N., Kenfoud H., Algethami F., Modwi A., Guesmi A., Assadi A.A., Khezami L. (2021). Application of Bi12ZnO20 Sillenite as an Efficient Photocatalyst for Wastewater Treatment: Removal of Both Organic and Inorganic Compounds. Materials.

[10] Li A., Deng H., Jiang Y., Ye C. (2020). High-Efficiency Removal of Cr(VI) from Wastewater by Mg-Loaded Biochars: Adsorption Process and Removal Mechanism. Materials.

[11] Guo J., Li J.-J., Wang C.-C. (2019). Adsorptive removal of Cr(VI) from simulated wastewater in MOF BUC-17 ultrafine powder. J. Environ. Chem. Eng..

[12] Siciliano A. (2016). Removal of Cr(VI) from Water Using a New Reactive Material: Magnesium Oxide Supported Nanoscale Zero-Valent Iron. Materials.

[13] Sočo E., Domoń A., Papciak D., Michel M.M., Cieniek B., Pająk D. (2022). Characteristics of the Properties of Absodan Plus Sorbent and Its Ability to Remove Phosphates and Chromates from Aqueous Solutions. Materials.

[14] Dovi E., Aryee A.A., Kani A.N., Mpatani F.M., Li J., Qu L., Han R. (2022). High-capacity amino-functionalized walnut shell for efficient removal of toxic hexavalent chromium ions in batch and column mode. J. Environ. Chem. Eng..

[15] Liu Y., Liu H., Shen Z. (2021). Nanocellulose Based Filtration Membrane in Industrial Waste Water Treatment: A Review. Materials.

[16] Liu C., Jin R.-N., Ouyang X.-k., Wang Y.-G. (2017). Adsorption behavior of carboxylated cellulose nanocrystal—Polyethyleneimine composite for removal of Cr(VI) ions. Appl. Surf. Sci..

[17] Gurgel L.V.A., de Melo J.C.P., de Lena J.C., Gil L.F. (2009). Adsorption of chromium (VI) ion from aqueous solution by succinylated mercerized cellulose functionalized with quaternary ammonium groups. Bioresour. Technol..

[18] Peng X., Yan Z., Cheng X., Li Y., Wang A., Chen L. (2019). Quaternary ammonium-functionalized rice straw hydrochar as efficient adsorbents for methyl orange removal from aqueous solution. Clean Technol. Environ. Policy.

[19] Dong Z., Zhao L. (2018). Covalently bonded ionic liquid onto cellulose for fast adsorption and efficient separation of Cr(VI): Batch, column and mechanism investigation. Carbohydr. Polym..

[20] Cumbal L., SenGupta A.K. (2005). Arsenic Removal Using Polymer-Supported Hydrated Iron(III) Oxide Nanoparticles:  Role of Donnan Membrane Effect. Environ. Sci. Technol..

[21] Qiu H., Liang C., Zhang X., Chen M., Zhao Y., Tao T., Xu Z., Liu G. (2015). Fabrication of a Biomass-Based Hydrous Zirconium Oxide Nanocomposite for Preferable Phosphate Removal and Recovery. ACS Appl. Mater. Interfaces.

[22] Qiu H., Liang C., Yu J., Zhang Q., Song M., Chen F. (2017). Preferable phosphate sequestration by nano-La(III) (hydr)oxides modified wheat straw with excellent properties in regeneration. Chem. Eng. J..

[23] Peng X., Yan Z., Hu L., Zhang R., Liu S., Wang A., Yu X., Chen L. (2020). Adsorption behavior of hexavalent chromium in aqueous solution by polyvinylimidazole modified cellulose. Int. J. Biol. Macromol..

[24] Mortazavian S., Saber A., Hong J., Bae J.-H., Chun D., Wong N., Gerrity D., Batista J., Kim K.J., Moon J. (2018). Synthesis, characterization, and kinetic study of activated carbon modified by polysulfide rubber coating for aqueous hexavalent chromium removal. J. Ind. Eng. Chem..

[25] Yang R., Aubrecht K.B., Ma H., Wang R., Grubbs R.B., Hsiao B.S., Chu B. (2014). Thiol-modified cellulose nanofibrous composite membranes for chromium (VI) and lead (II) adsorption. Polymer.

[26] Wu J., Wang X.-B., Zeng R.J. (2017). Reactivity enhancement of iron sulfide nanoparticles stabilized by sodium alginate: Taking Cr (VI) removal as an example. J. Hazard. Mater..

[27] Ma L., Islam S.M., Liu H., Zhao J., Sun G., Li H., Ma S., Kanatzidis M.G. (2017). Selective and Efficient Removal of Toxic Oxoanions of As(III), As(V), and Cr(VI) by Layered Double Hydroxide Intercalated with MoS_4_^2–^. Chem. Mater..

[28] Yazdi M.N., Dadfarnia S., Shabani A.M.H. (2021). Synthesis of stable S- functionalized metal-organic framework using MoS_4_^2-^ and its application for selective and efficient removal of toxic heavy metal ions in wastewater treatment. J. Environ. Chem. Eng..

[29] Peng X., Chen L., Liu S., Hu L., Zhang J., Wang A., Yu X., Yan Z. (2021). Insights into the Interfacial Interaction Mechanisms of p-arsanilic acid Adsorption on Ionic Liquid Modified Porous Cellulose. J. Environ. Chem. Eng..

[30] Chang C., Duan B., Zhang L. (2009). Fabrication and characterization of novel macroporous cellulose–alginate hydrogels. Polymer.

[31] Pour Z.S., Ghaemy M. (2015). Removal of dyes and heavy metal ions from water by magnetic hydrogel beads based on poly(vinyl alcohol)/carboxymethyl starch-g-poly(vinyl imidazole). RSC Adv..

[32] Jawad A., Liao Z., Zhou Z., Khan A., Wang T., Ifthikar J., Shahzad A., Chen Z., Chen Z. (2017). Fe-MoS_4_: An Effective and Stable LDH-Based Adsorbent for Selective Removal of Heavy Metals. ACS Appl. Mater. Interfaces.

[33] Ali J., Wang H., Ifthikar J., Khan A., Wang T., Zhan K., Shahzad A., Chen Z., Chen Z. (2018). Efficient, stable and selective adsorption of heavy metals by thio-functionalized layered double hydroxide in diverse types of water. Chem. Eng. J..

[34] Fu W., Chen H., Yang S., Huang W., Huang Z. (2019). Poly(diallyldimethylammonium-MoS4) based amorphous molybdenum sulphide composite for selectively mercury uptake from wastewater across a large pH region. Chemosphere.

[35] Gupta K., Huo J.-B., Yang J.-C.E., Fu M.-L., Yuan B., Chen Z. (2019). (MoS4)^2−^ intercalated CAMoS4⋅LDH material for the efficient and facile sequestration of antibiotics from aqueous solution. Chem. Eng. J..

[36] Wang Y., Gu Y., Xie D., Qin W., Zhang H., Wang G., Zhang Y., Zhao H. (2019). A hierarchical hybrid monolith: MoS_4_^2−^-intercalated NiFe layered double hydroxide nanosheet arrays assembled on carbon foam for highly efficient heavy metal removal. J. Mater. Chem. A.

[37] Ma L., Wang Q., Islam S.M., Liu Y., Ma S., Kanatzidis M.G. (2016). Highly Selective and Efficient Removal of Heavy Metals by Layered Double Hydroxide Intercalated with the MoS_4_^2–^ Ion. J. Am. Chem. Soc..

[38] Jawad A., Peng L., Liao Z., Zhou Z., Shahzad A., Ifthikar J., Zhao M., Chen Z., Chen Z. (2018). Selective removal of heavy metals by hydrotalcites as adsorbents in diverse wastewater: Different intercalated anions with different mechanisms. J. Clean. Prod..

[39] Xie L., Yu Z., Islam S.M., Shi K., Cheng Y., Yuan M., Zhao J., Sun G., Li H., Ma S. (2018). Remarkable Acid Stability of Polypyrrole-MoS4: A Highly Selective and Efficient Scavenger of Heavy Metals Over a Wide pH Range. Adv. Funct. Mater..

[40] Peng X., Luo Z., Xie H., Liang W., Luo J., Dang C., Wang A., Hu L., Yu X., Cai W. (2022). Removal of phenylarsonic acid compounds by porous nitrogen doped carbon: Experimental and DFT study. Appl. Surf. Sci..

[41] Liang X., Fan X., Li R., Li S., Shen S., Hu D. (2018). Efficient removal of Cr(VI) from water by quaternized chitin/branched polyethylenimine biosorbent with hierarchical pore structure. Bioresour. Technol..

[42] Foo K.Y., Hameed B.H. (2010). Insights into the modeling of adsorption isotherm systems. Chem. Eng. J..

[43] Wang J., Wang P., Wang H., Dong J., Chen W., Wang X., Wang S., Hayat T., Alsaedi A., Wang X. (2017). Preparation of Molybdenum Disulfide Coated Mg/Al Layered Double Hydroxide Composites for Efficient Removal of Chromium(VI). ACS Sustain. Chem. Eng..

[44] Wang J., Wang X., Zhao G., Song G., Chen D., Chen H., Xie J., Hayat T., Alsaedi A., Wang X. (2018). Polyvinylpyrrolidone and polyacrylamide intercalated molybdenum disulfide as adsorbents for enhanced removal of chromium(VI) from aqueous solutions. Chem. Eng. J..

[45] Li Y., Zhu H., Zhang C., Cheng M., He H. (2018). PEI-grafted magnetic cellulose for Cr(VI) removal from aqueous solution. Cellulose.

[46] Wang H., Yuan X., Wu Y., Chen X., Leng L., Wang H., Li H., Zeng G. (2015). Facile synthesis of polypyrrole decorated reduced graphene oxide–Fe_3_O_4_ magnetic composites and its application for the Cr(VI) removal. Chem. Eng. J..

[47] Qiu J., Liu F., Cheng S., Zong L., Zhu C., Ling C., Li A. (2018). Recyclable Nanocomposite of Flowerlike MoS_2_@Hybrid Acid-Doped PANI Immobilized on Porous PAN Nanofibers for the Efficient Removal of Cr(VI). ACS Sustain. Chem. Eng..

[48] Zhu K., Chen C., Xu H., Gao Y., Tan X., Alsaedi A., Hayat T. (2017). Cr(VI) Reduction and Immobilization by Core-Double-Shell Structured Magnetic Polydopamine@Zeolitic Idazolate Frameworks-8 Microspheres. ACS Sustain. Chem. Eng..

[49] Dong Z., Zhao L. (2018). Surface modification of cellulose microsphere with imidazolium-based ionic liquid as adsorbent: Effect of anion variation on adsorption ability towards Au(III). Cellulose.

[50] Zhang Y., Wang H., Sun N., Chi R. (2018). Experimental and computational study on mechanism of dichromate adsorption by ionic liquid-bonded silica gel. Sep. Purif. Technol..

[51] Zhang X., Fu W., Yin Y., Chen Z., Qiu R., Simonnot M.-O., Wang X. (2018). Adsorption-reduction removal of Cr(VI) by tobacco petiole pyrolytic biochar: Batch experiment, kinetic and mechanism studies. Bioresour. Technol..

