# Supplementary material for: Selective Removal of Hexavalent Chromium by Novel Nitrogen and Sulfur Containing Cellulose Composite: Role of Counter Anions
Source: Materials (Basel). 2022 Dec 25;16(1):184. doi: 10.3390/ma16010184 (PMC9821927; doi:10.3390/ma16010184)
Supplement: Supplementary file 1 [file materials-16-00184-s001.zip › materials-2084000-supplementary.pdf]

materials

Supporting information

Selective Removal of Hexavalent Chromium by Novel Nitrogen and Sulfur Containing Cellulose Composite: Role of Counter Anions

Xiong Peng [1,2], Shujun Liu [2], Zhijia Luo [1], Xiwen Yu [3] and Wanwen Liang [1,*]

[1] School of Chemistry and Chemical Engineering, Guangzhou University, Guangzhou, 510006, China
[2] School of Chemistry and Chemical Engineering, South China University of Technology, Guangzhou 510640, China
[3] Institute of Biological and Medical Engineering, Guangdong Academy of Sciences, Guangzhou 510316, China
* Correspondence: wanwen.liang@foxmail.com

The kinetic models were described by pseudo-first order kinetic model:

$$Q_t = Q_e(1 - e^{-k_1 t}) \tag{S1}$$

and the pseudo-second order kinetic model:

$$Q_t = \frac{k_2 Q_e^2 t}{1 + k_2 Q_e t} \tag{S2}$$

where Q_t (mg/g) denotes the removal capacity at time t (min); Q_e is the removal capacity at equilibrium; k_1 and k_2 represent the pseudo-first-order and pseudo-second-order rate constants, respectively.

The adsorption isothermal models were described by Langmuir and Freundlich isotherm model. The Langmuir isotherm model can be expressed as:

$$Q_e = \frac{K_L Q_{max}}{1 + K_L c_e} \tag{S3}$$

and the Freundlich isotherm model can be expressed as:

$$Q_e = K_f C_e^{\frac{1}{n}} \tag{S4}$$

where Q_e (mg/g), and C_e (mg/L) are the uptake capacity and the adsorption equilibrium concentration of organoarsenic compounds, separately. Q_{max} (mg/g) is theoretical maximum uptake capacity. K_L and K_f are Langmuir and Freundlich adsorption constant at equilibrium, respectively; n is used to describe adsorption intensity.

The Sips isotherm model is the combination of Langmuir and Freundlich models, and expressed as:

$$Q_e = \frac{K_s Q_{max} C_e^{\frac{1}{n}}}{1 + K_s C_e^{\frac{1}{n}}} \tag{S5}$$

where K_s is the Sips constant, which is related to the adsorption energy. The physical meanings of other parameters are the same as those of Langmuir and Freundlich models.

The thermal dynamic parameters, ΔH (kJ/mol), ΔG (kJ/mol), and ΔS (kJ/mol) were calculated by the equations below:

$$\ln K_d^0 = \ln \frac{1000 Q_e}{C_e} = \frac{\Delta S}{R} - \frac{\Delta H}{RT} = -\frac{\Delta G}{RT} \tag{S6}$$

where K_d^0 is the dimensionless distribution coefficient, Q_e (mg/g) and C_e (mg/L) are the same as mentioned above.

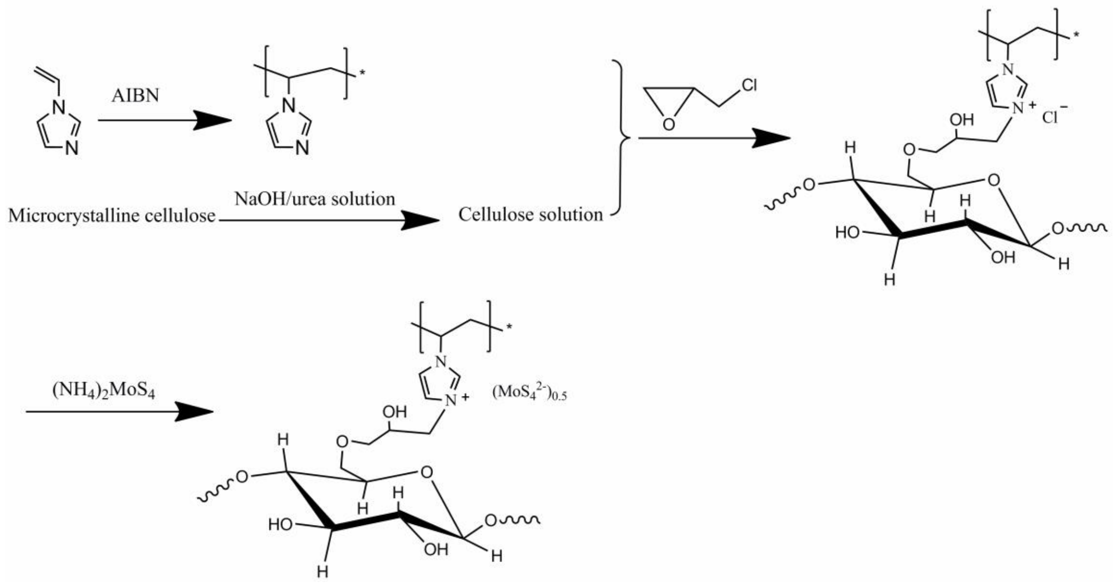

Figure S1. The synthesis routine of MPS composite.
